# Qualitative Behavioural Assessment of bonobo emotional expressivity across observer groups and zoo housing environments

**DOI:** 10.1017/awf.2024.29

**Published:** 2024-05-30

**Authors:** Daan W Laméris, Marina Salas, Marcel Eens, Lisa Gillespie, Nicky Staes, Jonas RR Torfs, Jonas Verspeek, Hilde Vervaecke, Samantha J Ward, Jeroen MG Stevens

**Affiliations:** 1 Behavioural Ecology and Ecophysiology, University of Antwerp, Universiteitsplein 1, 2610 Wilrijk, Belgium; 2 Antwerp Zoo Centre for Research and Conservation, Royal Zoological Society of Antwerp, Koningin Astridplein 26, 2018 Antwerp, Belgium; 3 Twycross Zoo, East Midland Zoological Society, Burton Rd, Atherstone CV9 3PX, UK; 4 Salto Research Group, Agro-and Biotechnology, Odisee University of Applied Sciences, Hospitaalstraat 21, 9100 Sint-Niklaas, Belgium; 5 School of Animal, Rural and Environmental Sciences, Nottingham Trent University, Southwell, NG25 0QF, UK

**Keywords:** Animal welfare, emotion, Fixed List, Free Choice Profiling, primate, QBA

## Abstract

Human evaluation of animal emotional expressivity can inform animal welfare. Qualitative Behavioural Assessment (QBA) has been applied to domesticated and some non-domesticated animals, but its use in primates is limited despite their emotional expressivity. We aimed to develop and apply a QBA for bonobos (*Pan paniscus*) through two consecutive studies. We applied Free Choice Profiling (FCP) and the Fixed List methodology, respectively, in Study 1 and 2, and invited students and bonobo experts to rate video clips of zoo-living bonobos of different sexes and age classes, and before and after moving to a new enclosure. In Study 1, students described dimension 1 as ranging from ‘quiet/calm’ to ‘angry/active’ and dimension 2 from ‘sad/anxious’ to ‘happy/loving’. Experts described dimension 1 ranging from ‘quiet/relaxed’ to ‘nervous/alert’ and dimension 2 from ‘nervous/bored’ to ‘playful/happy’. Using a fixed list of descriptors, informed by findings from Study 1, students in Study 2 described dimension 1 as ranging from ‘quiet/calm’ to ‘agitated/frustrated’, and dimension 2 from ‘sad/stressed’ to ‘happy/positively engaged’. Experts described dimension 1 as ranging from ‘quiet/calm’ to ‘active/excited’, and dimension 2 from ‘sad/bored’ to ‘happy/positively engaged’. Students scored adults as more ‘calm/quiet’ and experts scored subadults as more ‘happy/positively engaged’. Additionally, experts in Study 2 rated bonobos as more ‘active/excited’ in their new enclosure. Reliability was moderate to good for the dimensions. Additionally, animal-directed empathy of observers influenced QBA scores. This is the first time, FCP has been successfully used as a method to study primate emotional expressivity. Our findings show the promise of employing QBA in primate studies and in industry, with validation of additional metrics to enable its use for welfare-monitoring purposes.

## Introduction

Measuring animal emotional states has been of interest for many scientific fields, including animal welfare science (Mellor 2012) and comparative psychology (Kret *et al.* 2022). As a result of this widespread interest, numerous measures have been developed to estimate emotions in animals. Emotional states correlate with behavioural, physiological and/or cognitive responses, of which a number can be identified and quantified. Behaviour remains one of the most commonly utilised parameters to assess welfare (Dawkins 2004; Binding *et al.* 2020). Anthropomorphism is, however, often considered a methodological concern for behavioural observations that should be avoided (de Waal 1999; Williams *et al.* 2020). Nonetheless, judgements that are quantified and validated can provide practical and scientific advantages (Meagher 2009). A prominent approach herein is the Qualitative Behavioural Assessment (QBA) method, developed in the early 2000s to evaluate animal welfare, which focuses on the ‘whole-body’ expressive qualities of an animal (Wemelsfelder *et al.* 2000). Instead of focusing on separate facets of information and measuring isolated components of the animal’s behaviour, QBA is a holistic and integrative approach to examine how animals respond to the environment and how they deal with it (Wemelsfelder *et al.* 2001). Two main QBA approaches currently exist: the Free Choice Profiling (FCP) method, originating from food sciences, allows raters to use their own descriptive terminology to score animal emotional expressions, and the Fixed-List method which provides raters with a list of predetermined descriptors used by all raters. QBA shares similarities with rater-based animal personality scores as similar terms are used, but allows for the temporal variation of emotional states when repeatedly measured (Wemelsfelder *et al.* 2000).

QBA was initially developed as a welfare indicator for farm animals to supplement other quantitative measures (Wemelsfelder *et al.* 2000). A major benefit of QBA is that assessments can be done rapidly, on-site (through live observations) or off-site (using video footage), on the individual or at a group-level. High inter- and intra-observer reliability, and validation against other behavioural and physiological indicators of welfare states have proven the value of QBA as an additional welfare measure (Stockman *et al.* 2011; Wickham *et al.* 2015; Carreras *et al.* 2016; Skovlund *et al.* 2023).

QBA is based on the human ability to interpret the expressive qualities or demeanour of an animal, and this may be modulated by several of the observer’s characteristics. This can include the level of experience with the species (Duijvesteijn *et al.* 2014), which for non-domesticated species may present a challenge as the shared co-evolution and enhanced familiarity with domesticated species facilitates our interpretation and responses to these animals (Westbury & Neumann 2008; Prguda & Neumann 2014). Another factor that may influence how humans perceive animals is their empathy with animals, which can be a concern for the generalisability of observer judgements (Meagher 2009). The ability of humans to perceive and assess emotional states of others is influenced by their level of empathy, which varies among individuals and as such may influence their judgements. Nonetheless, QBA has been shown to be a valuable additional tool to assess the welfare of non-domesticated species, for example in zoo settings or wildlife rescue centres (Rose & Riley 2019). Recently, QBAs have been gaining momentum for such species, and a number of studies developed and tested QBAs for non-domesticated species, including giraffes (*Giraffa camelopardalis*: Patel *et al.* 2019), elephants (*Loxodonta africana* and *Elephas maximus*: Yon *et al.* 2019; Webb *et al.* 2020; Pollastri *et al.* 2021), brown bears and polar bears (*Ursus arctos*: Stagni *et al.* 2022; *Ursus maritimus*: Skovlund *et al.* 2023) and dolphins (*Tursiops truncatus*: Warner *et al.* 2022). Non-human primates (from now on ‘primates’) have so far received limited attention, with only one study applying a QBA following the Fixed-List method (Gartland *et al.* 2022), while no previous studies have used FCP to study primate emotional expressivity.

Primates may be particularly interesting for QBA as emotional expressions play a pivotal role in maintaining and regulating social relationships in many primate societies. One noteworthy example is the bonobo (*Pan paniscus*), a great ape species known for their pronounced emotional expressiveness, which serves a pivotal role in regulating their social dynamics. For example, distinct facial expressions and vocalisations are produced during positive and negative social interactions (e.g. play or bared-teeth faces: de Waal 1988; Demuru *et al.* 2015) which signal information about the sender’s emotional state and present highly salient visual signals driving attentional mechanisms for the rapid detection of such signals (Kret *et al.* 2016; Laméris *et al.* 2022a; van Berlo *et al.* 2023). Other behaviours, such as certain self-directed behaviours, have been shown to be reliable indicators of negative emotional arousal in bonobos (Laméris *et al.* 2022b). Some of these expressions exhibit continuity among species, including humans, and homologous traits can be identified, whilst for other expressions this is not the case (Kavanagh *et al.* 2022).

Observer-based scores have previously been applied to different primate species to address emotional states and generally found good reliability between expert observers (e.g. people with behavioural observation experience or caretakers) (Stevenson-Hinde *et al.* 1980; Weiss *et al.* 2002; King & Landau 2003; Robinson *et al.* 2016), but the use of QBA remains limited (Gartland *et al.* 2022). On one hand, their phylogenetic proximity and physical similarity to humans may facilitate human recognition and perception of primate body language and gestures (Graham & Hobaiter 2023). Additionally, this similarity may enhance our empathetic attitudes towards primates (Miralles *et al.* 2019), which can subsequently influence how we judge their emotions. On the other hand, due to the high degree of expressive variation and the homologous nature of some traits between primates, correctly identifying and recognising these expressions may be a challenge for the general public, compared to primate experts (Foley 1935; Waller *et al.* 2007). Although welfare assessments carried out by the general public could be an informative asset (Freire *et al.* 2021), some level of experience with the species is expected to be necessary for the reliable use of QBA in non-human primates.

The current study aimed to develop and explore the use of a QBA for bonobos. As such, we completed two studies. In Study 1, we applied Free Choice Profiling (FCP) to examine the terminology used by experts and non-experts to describe bonobo emotional expressivity. In Study 2, we built on the outcomes of Study 1 and utilised the most commonly used terms to develop a list of fixed terms that can be further used to assess emotional states in bonobos. For both studies, we additionally sought to investigate if: (a) observers, who differed in their level of experience with bonobos, scored the expressivity of bonobos differently; (b) animal-directed empathy levels of observers influenced their QBA scoring; and (c) observers perceived differences in the bonobos’ expressivity based on contextual or individual factors related to the bonobos. As a specific contextual factor, we considered housing condition, as we recorded videos during a period before and after the bonobos moved to a new enclosure within a zoo. The individual factors of the bonobos that we examined were sex and age class.

## Materials and methods

### Ethical considerations

This study was reviewed by an independent ethical committee, the Social Sciences and Humanities Ethics Advisory Committee (EA SHW) of the University of Antwerp, who issued a favourable opinion on 07-03-2022 (#SHW_22_026). All human participants received an information and consent sheet and provided written consent. The recording of the video footage of the bonobos was approved by the Twycross Zoo Research Committee in May 2021. The bonobos were housed in an EAZA-accredited institution and managed according to the Bonobo Best Practice Guidelines (Stevens 2020).

### Study animals and housing conditions

Ten bonobos were selected from a group of twelve individuals, housed in Twycross Zoo (UK). Subjects were selected with the aim of covering both sexes and different age categories in as balanced a way as possible. As such, four adult females, four adult males and two subadult males (< 7 years old) were the subjects of the study. Subjects ranged in ages from 5.6 to 36.3 years of age at the time of filming (mean [± SD] = 19.1 [± 8.2] years). The bonobos were housed in two subgroups whereby the individuals within the groups were changed regularly in accordance with the needs of the animals and their fission-fusion societal management system. They received regular provision of targeted and group scatter feeds throughout the day, had access to water *ad libitum* and were provided with enrichment and browse on a daily basis. Between 14–22 September 2021, the bonobos were moved to a new enclosure. The original enclosure consisted of two separate areas within the same building and were similar in size (2 × 52.8 m^2^). The two enclosures were able to be connected via automatic sliding doors. Indoor areas contained large permanent climbing structures, with wooden beams, dynamic webbing material, nesting platforms, and off-show bed areas. There was a single, shared outdoor space (547 m^2^), with access for each group rotated every 24 h. The outdoor area included further climbing structures, hiding areas and a drinking pond with fresh running water, and was visible to the public. The new enclosure, despite being larger in size (2 × 54.3 m^2^), provided a very similar environment with the same husbandry procedures as the pre-move enclosure. The main distinction being that both groups always had full access to an outdoor area (433 m^2^ and 211 m^2^), as opposed to alternating access every 24 h.

### Video footage

Video footage was recorded during two periods; the first spanning five days between July and September 2021 in the old bonobo enclosure and the second lasting four days in November 2021, following the relocation of the bonobos to their new enclosure. The videos were captured multiple times per day randomly between 0900 and 1600h via mobile phones (iPhone, Cupertino, USA, Samsung, Suwon, South Korea) or hand-held cameras (Canon Legria HF R88, Tokyo, Japan). During each recording moment, the aim was to collect footage of each focal animal, unless they were out of sight. We filtered out low-quality videos, or when bonobos did not stay in view for at least 30 s. In total, we retained 227 videos, ranging from 30 to 120 s in length, resulting in approximately 270 min of footage. From these videos, we selected the first and/or last 30 s in which one of the ten focal animals was fully visible to create a library of 30-s video clips of random snapshots of the focal individual’s demeanour (mean [± SEM] = 27.8 [± 2.5] video clips per bonobo; range = 19–45; Table S1; see Supplementary material). The focal animal was later identified in each video using a white arrow to simplify identification for the group of observers that were unfamiliar with the individuals. This video library was utilised in both Study 1 and 2 to select video clips to present to the observers.

### Survey and Animal Empathy Scale (Paul 2000)

Prior to participating in the QBA sessions, we asked the participants of Study 1 (n = 26) and 2 (n = 49) to fill out a survey consisting of two parts. The first part focused on demographic information, such as age, previous/current pet ownership, how often they visited zoos, and previous/current experience working with animals, and whether this was specific to primates. The distribution of this data is presented in Table S2 (see Supplementary material). We did not further explore the effect of pet ownership and professional experience working with animals, as there was little variation based on these variables. The second part of the survey aimed to establish a level of empathy with animals for each of the participants. We asked the participants in both studies to complete the Animal Empathy Scale (Paul 2000). This survey contains statements regarding the way people feel about animals and invites participants to score each statement on a nine-point Likert scale (ranging from ‘strongly disagree’ to ‘strongly agree’). Six observers from the students’ group (Study 1: n = 1; Study 2: n = 5) did not fully complete the survey and were therefore not included in the empathy analysis.

Following the methodology of Cornish *et al.* (2018), we conducted a Ward’s Hierarchical Clustering analysis using Euclidian distance to investigate how participants’ responses to the statements of the Animal Empathy Scale clustered together in both Study 1 and 2. These analyses revealed two distinct clusters, classifying the statements as either ‘empathic’ or ‘apathic’ (see Table S3; Supplementary material). Based on these clusters, we calculated empathy ratio scores for individual observers by dividing the average of the scores of the statements within the ‘empathic’ cluster by the average of the scores of the statements in the ‘apathic’ cluster. Values above 1 indicate that participants agreed more with ‘empathic’ statements, while values below 1 indicate a higher agreement with ‘apathic’ statements. These empathy ratio scores were subsequently used to investigate the relationship between empathy for animals in Study 1 and 2 and how observers scored along the constructed dimensions.

### Study 1: Free Choice Profiling

#### Observers

In Study 1, two groups of observers participated. The first group consisted of 17 students (age range: 18–35 years) who were enrolled in a behavioural biology course at Odisee University of Applied Sciences, Belgium and had some prior experience with QBA. The second group comprised nine animal experts (age range: 28–64 years) who had previous experience working on topics such as animal welfare or bonobo behaviour. Some of the experts were familiar with QBA. To accommodate logistics, we organised two sessions in April 2022. The first involved all the students and five experts, while the remaining four experts participated in a separate session. During both sessions, the observers were seated behind a computer or laptop and each began with an instructional period lasting approximately 45 min. During this instruction, an explanation was offered as what QBA consists of and how it is used. Next, we explained that the objective of this study was to explore whether QBA could be used to assess the welfare of zoo-housed bonobos. Finally, Phases 1 and 2 (described below) were outlined via a practice video. Observers were specifically instructed to focus upon the bodily expressions of the focal animal, without them receiving additional information regarding bonobo behaviour. We did not instruct the participants to pay attention to differences between housing conditions, sexes, or age classes, which could potentially be inferred from visual cues. Furthermore, we explained to the observers that there were no ‘correct’ or ‘incorrect’ answers and explicitly instructed them to complete the QBA on their own. Throughout the sessions observers were requested to discuss neither their terminology nor the videos.

#### Phase 1 – Term generation

The goal of Phase 1 was for the observers to generate their own terminology describing the range of bonobo emotional expressivity. We applied FCP for the initial term generation which is an integrative methodology that allows observers to independently generate their own descriptive terminology that, in their opinion, best describes the animal’s emotional expressivity (Wemelsfelder *et al.* 2001). FCP consists of two phases. In Phase 1, the observers viewed 20 × 30-s video clips of bonobos on a computer or laptop. These videos were selected from our video library to cover a wide range of behavioural expressions in bonobos (Table S4; Supplementary material). After each clip, the observers had 2 min to list the adjectives that most aptly described the expressive qualities of the bonobos on paper. They were instructed to generate as many descriptors as they could come up with and allowed to re-use terms for subsequent videos. Observers were also permitted to write down terms in their native language (i.e. Dutch). After all the clips were viewed, the observers were instructed to create a list of unique terms that they had used. Two researchers (DWL and JMGS) then checked these lists and deleted terms that described what the animals were doing (e.g. walking, feeding), as well as terms that were given both in their positive and negative form, by keeping only the positive form (e.g. keeping only ‘happy’ out of ‘unhappy’ and ‘happy’). Moreover, we changed the order of the terms to increase the contrast between them. For this publication, terms were translated into English and two independent people (fluent in both English and Dutch) translated these back to Dutch as double control (Table S5; Supplementary material).

#### Phase 2 – Rating procedure

After a break, the observers received their checked, final list of terms and continued with Phase 2. The purpose of this phase was for the observers to use their own terminology on a quantitative basis to score bonobo expressivity. In contrast to previous studies where ratings were recorded onto paper scoring sheets, a web-based visual analogue scale (VAS) was implemented for more reliable and efficient data processing (Couper *et al.* 2006). The observers were provided with a link to a Qualtrics survey (Qualtrics, Provo, UT, USA), to which they were asked to transfer their final list of terms. In this phase, a new set of 20 video clips was presented to the observers, which had been randomly selected from our video library to cover one video for each of the ten individuals in the old and new enclosure. However, an foreseen error meant that for one individual we selected two clips in the new enclosure, resulting in a total of nine clips from the old enclosure and eleven from the new one. The video presentation was integrated within Qualtrics, and we configured the settings to automatically associate each term from the personal list with a VAS to cover a range of 0 to 100 points. The VAS had anchors labelled as ‘minimum’ and ‘maximum’, with the left end of the scale (i.e. ‘minimum’) meaning that the expressive quality indicated by the term was completely absent, and the right end of the scale (i.e. ‘maximum’) indicating that the term was fully expressed. Through a slider bar, the observers could click a point on the VAS to give a score for each term for each of the 20 videos. We set no default location of the slider, and it only became visible when the rater clicked at a point somewhere on the VAS. No number of points were set along the visual analogue slider and neither was numeric reference of the score provided, in an attempt to resemble analogue VAS as much as possible.

#### Statistical analysis

The 26 observers individually assigned quantitative scores to their own terms for each of the 20 videos in Phase 2. Data were analysed separately for the students and experts using Multiple Factor Analysis (MFA), which is an extension of Principal Component Analysis (PCA) designed to analyse multiple data-sets with different variables collected from the same set of observations. MFA belongs to the family of multi-table methods and is similar to the widely used Generalised Procrustes Analysis (GPA) (Wemelsfelder *et al.* 2000). Both procedures are freely accessible in the *FactoMineR* package (Lê *et al.* 2008) in Rstudio (R Core Team 2020), however, MFA has the statistical advantage over GPA of being an eigendecomposition technique that does not require multiple iterations to reach a consensus.

A detailed review of MFA is provided by Abdi *et al.* (2013) but, in brief, MFA aims to: (a) analyse multiple data-sets with different variables on the same observations; (b) provide a set of compromise factor scores; and lastly (c) project the original data onto the compromise, which is a common representation of the observations (similar to the ‘consensus profile’ in GPA) that allows you to analyse communalities and discrepancies between observations. To achieve this, MFA first standardises each of the data-sets so that the first principal component of each table has a similar length, which is measured by the first singular value. Repeated measures are accounted for by structuring the data into one matrix where rows correspond to the video clips and columns to each of the descriptors given by the observers. Columns are then structured by identifying grouping variables (i.e. observer identity). Next, a non-normalised PCA is performed on the sequence of normalised data tables to obtain common representation of the observations, referred to as the compromise. This compromise consists of a set of principal components (or dimensions), which can be ordered by the amount of variance that each dimension explains. The observations can be plotted along these dimensions, and their respective locations can be expressed in their co-ordinates (i.e. factor scores). The distance between the observations within this compromise expresses the similarities between the observations. This can be further broken down to the observations of each individual data table, which are referred to as partial factor scores.

MFA additionally calculates a similarity matrix between each possible pair of observers. We performed a Principal Coordinate Analysis (PcoA) on these similarity values to estimate the centre of distribution for each of the observers and calculate 95% confidence regions. Observers outside these regions are considered potential outliers. The observer plots depicting these regions can be found in Figure S1 (see Supplementary material), revealing three potential outliers in the student group. However, we decided to retain these observers as we had no discernible reason to believe their ratings to be invalid. To assess whether the results of the MFA truly reflected patterns in the detection of emotional expressions of bonobos or if they were merely statistical artifacts, we ran a permutation test on the eigenvalues of the first dimensions. By running the MFA 500 times, using permuted datasets, we were able to calculate 95% confidence intervals for these ‘random’ eigenvalues. Observed eigenvalues for dimensions from our true data-set that were above these confidence intervals would indicate that the variance explained by the compromise was a meaningful feature of the data-set, and not a statistical artifact. Inter-observer reliability of the final dimensions underwent further assessment using Intra-Class Correlations (ICC) via a two-way mixed model and a consistency definition (Koo & Li 2016).

MFA transformed the different configurations into one multidimensional compromise profile which is defined purely in terms of its geometrical properties without any semantic connotations. To assign semantic meaning to the dimensions of the compromise profile, we examined the loadings between all the terms generated by the observers and the principal dimensions. Here, the stronger a term loads onto a dimension, the more that term can be considered a representative descriptor of that dimension. For dimension 1, we used terms with loadings lower than –0.7 and higher than 0.7. For dimension 2, we kept terms with loadings lower than –0.4 and higher than 0.4. We then counted how many times each descriptor was above and below the dimension-specific threshold and used those terms that occurred most frequently to describe the two dimensions.

We were additionally interested if observers perceived differences in the emotional expressions of the bonobos depending on contextual (housing condition [old enclosure or new enclosure]) or focal animal factors (age class [subadult or adult], sex [female or male]). We analysed the location of the factor scores per observer per video clip along the dimensions in separate linear mixed models against housing condition, age class and sex as predictor variables. Each model included a random intercept for observer ID and video clip. Focal animal ID was considered as additional random intercept but decreased the model fit. In an additional linear mixed model, we tested the partial factor scores against the observers’ animal empathy scores as predictor variable, separately for the student and expert group.

MFA was performed using the *FactoMineR* package (Lê *et al.* 2008), and Linear Mixed Models were performed using the *lme4* package (Bates *et al.* 2015) in RStudio version 1.3.1073 (R Core Team 2020).

### Study 2: Fixed List procedure

#### Observers

For Study 2, we invited 44 new students (age range: 18–44 years) from the same course as the students from Study 1 and reinvited the experts who had also participated in Study 1. Four experts (age range: 25–44 years) were able to participate in Study 2, and one additional expert, who had not participated in Study 1, rated the videos.

#### Rating procedure

We randomly selected 40 × 30-s videoclips from our video library that were equally divided across the ten bonobos and the old and new enclosure. Each observer viewed and rated four clips per individual bonobo, two for each housing condition.

Study 2 was carried out online via a live connection. Prior to starting the assessment, the observers received a ~45-min long instruction, including a practice video, which sought to explain the goal of the study and QBA process were explained. The terms and their definitions were also explained and discussed.

#### Selection of terms

The observers in Study 1 came up with 170 unique terms that were then subjected to MFA, resulting in two dimensions which we will describe in more detail in the *Results.* From these 170 terms, we selected 21 that were most occurrent, showed high positive and negative loadings in the two dimensions and covered a range of expressive qualities. ‘Lethargic’, ‘Positively engaged’ and ‘Indifferent’ were subsequently added, based on existing literature and author discussions (Table 1). The specific terms selected and their respective definitions meant it was occasionally necessary to describe one term using another.

**Table 1.** Final version of Fixed List of terms selected for use in Study 2 and their characterisations

Term	Description
Active	Bonobo radiates energy and strength, in an excited manner.
Agitated	The bonobo appears restless and edgy.
Anxious	The bonobo appears worried, unable to respond well to its surroundings, afraid.
Bored	The bonobo appears disinterested, passive, tired. May be looking to do something, in an unmotivated way.
Calm	The bonobo appears peaceful, without worries, and behaves relaxed and carefree.
Curious	The bonobo appears exploratory and proactive in searching its environment.
Excited	Positively restless in response to external stimuli, euphoric, exuberant, enthusiastic.
Focused	Bonobo appears concentrated on an action and pays attention to it.
Frustrated	Bonobo seems to lack fulfilment/satisfaction, is unable to achieve a goal.
Happy	The bonobo looks cheerful, unconcerned and shows joy.
Indifferent^a^	The bonobo does not appear to be concerned with other factors around it.
Irritated	The bonobo appears irritable and reacts negatively to a peer or environment, possibly with rejection.
Lethargic^a^	The bonobo looks tired and sluggish. Lacking strength and energy, it shows little movement, and every movement is slow and laborious.
Lively	The bonobo seems active, enthusiastic, full of life and energy. Regardless of physical activity, the bonobo shows positive energy and strength.
Nervous	The bonobo appears worried, highly reactive, and excited. They are alert and can be restless.
Playful	The bonobo makes lively movements, together or alone for fun, expressing pleasure, happiness, and amusement.
Positively engaged^a^	The bonobo performs activities in a focused, constructive manner. The bonobo does not seem distracted by others or the environment.
Quiet	Peaceful and carefree. The bonobo behaves unconcerned.
Relaxed	At rest, without tension. They seem unconcerned and at ease.
Sad	Bonobo appears cheerless and gloomy.
Satisfied	The bonobo appears pleased and content.
Self–confident	Bonobo shows assertiveness and behaves like this towards other animals in the environment.
Social	Bonobo interacts actively with others, is prepared to interact, and shows social behaviour.
Stressed	The bonobo appears tense, overstrained and edgy.

a Terms that have been added from the literature.

### Statistical analysis

Prior to running our statistical analyses, we examined if our data were suited for factor analysis by means of the Kaiser-Meyer-Olkin (KMO) test. In brief, the KMO test measures the proportion of variance among variables that might be shared. Here, lower values indicate shared correlations which are undesired for factor analysis. We handled a threshold of > 0.6 for including descriptors in our analysis. We employed Dual Multiple Factor Analysis (DMFA), which is an extension of MFA suitable when the same variables are measured, but allows for the partitioning of observers in groups, i.e. students and experts. The key benefit of DMFA over the commonly applied PCA, is its ability to handle multiple data-sets (i.e. individual observer data-sets) and to standardise the entered data per group (i.e. students and experts) (Lê & Pagés 2010). This standardisation enables direct comparison of the way the different observer groups use the fixed terms, and hence perceived the bonobos’ emotional expressivity. Given our interest in identifying and examining these potential differences, we considered DMFA more suitable than PCA. DMFA was performed using the *FactoMineR* package (Lê *et al.* 2008). As in Study 1, we used Linear Mixed Models (Bates *et al.* 2015) to test the effect of contextual (housing condition [old enclosure or new enclosure]), focal animal factors (age class [subadult or adult], sex [female or male]) and animal-directed empathy. Inter-observer reliability for the dimensions and individual descriptors was assessed using ICCs via a two-way mixed model and a consistency definition (Koo & Li 2016).

## Results

### Animal-directed empathy

Across both studies, participants (n = 65) had a mean (± SD) empathy ratio score of 2.76 (± 1.19); range = 1.19–6.93, meaning that all participants had some level of empathy for animals. There was no difference in mean (± SEM) empathy scores between the students (2.71 [± 0.15]) and experts (3.02 [± 0.50]); (χ² = 0.544, df = 1; *P* = 0.461).

### Study 1: Free Choice Profiling

#### Interpretation of the consensus profile

The 26 observers collectively produced a total of 640 (170 unique) terms to describe the expressive qualities of the bonobos, with an average of 24.6 terms (ranging 14–30) per observer. For the two groups specifically, the students came up with an average of 23.1 terms (ranging 14–30) and the experts with an average of 27.6 terms (ranging 14–30).

Based on the analysis of the permuted confidence intervals, we identified that the first two dimensions explained a statistically relevant proportion of the variance in the compromise profile, which corresponds to 32.8% in the student compromise (dimension 1: 22.2%, dimension 2: 10.6%), and 38.8% in the expert compromise (dimension 1: 25.6%, dimension 2: 13.1%). Intra-class correlations were higher for dimension 1 compared to dimension 2, and slightly higher for experts (0.75 and 0.50, respectively) than for students (0.73 and 0.42, respectively).

In Table 2, we list the strongest loading terms on dimension 1 and 2, where for dimension 1, terms with loading values greater than 0.7 or lower than –0.7 are considered, and for dimension 2, terms with loading values greater than 0.4 or lower than –0.4 are included. Based on the frequency in which these terms have been used across the observers, we described the dimensions for the different observer groups. For the students, dimension 1 was described as ranging from ‘quiet/calm’ to ‘angry/active’, and dimension 2 as ‘sad/anxious’ to ‘happy/loving’. For the expert group, dimension 1 was described as ranging from ‘quiet/relaxed’ to ‘nervous/alert’, and dimension 2 from ‘nervous/bored’ to ‘playful/happy’.

**Table 2.** Terms used by the observers (17 students and 9 experts) in Study 1 with strong factor loadings on the Multiple Factor Analysis dimensions. For dimension 1, terms with loading values greater than 0.7 or less than –0.7 are displayed. For dimension 2, terms with loading values greater than 0.4 or less than –0.4 are displayed. Figures in brackets indicate the number of times these terms met this criterion

	Negative loading	Positive loading
Students		
Dimension 1	Quiet (8), Calm (6), Relaxed (2), Bored, Withdrawn, Unimpressed, Carefree	Angry (8), Active (8), Frustrated (6), Irritated (4), Stressed (2), Restless (2), Playful (2), Tense, Panicky, Panicking, Noisy, Nervous, Hunted, Hostile, Fierce, Explosive, Excited, Dominant, Defensive, Curious, Busy, Anxious, Alert, Agitated, Aggressive, Afraid, Alert
Dimension 2	Sad (8), Anxious (7), Nervous (7), Bored (5), Stressed (3), Insecure (3), Frustrated (2), Lonely (2), Restless (2), Afraid (2), Worried (2), Compulsive, Attentive, Unhappy, Concerned, Tense, Irritated, Panicky, Evasive, Submissive, Confused, Withdrawn, Shy, Curious, Uncomfortable, Reluctant, Intimidated, Absent, Happy, Playful, Comfortable, Observant, Suspicious, Excluded, Content	Happy (5), Loving (3), Greedy (2), Relaxed (2), Inattentive, Caring, Playful, Protective, Helping, Loyal, Content, Sweet
Experts		
Dimension 1	Quiet (5), Relaxed (4), Calm (3), Satisfied	Nervous (4), Alert (3), Excited (2), Active (2), Intense (2), Playful (2), Frustrated (2), Attention–seeking, Agitated, Curious, Stressed, Tense, Altered, Challenging, Adventurous, Exploring boundaries, Brusque, Reckless, Insecure, Enthusiastic, Mobile, Impulsive, Worried, Annoyed
Dimension 2	Nervous (8), Bored (4), Anxious (3), Stressed (3), Tense (2), Frustrated, Upset, Timid, Dissatisfied, Difficult, Undecided, Angry, Shocked, Worried, Agitated, Afraid, Drowsy, Submissive, Attentive, Concerned, Waiting, Curious, Uncomfortable, Insecure	Playful (6), Happy (5), Challenging (3), Jealous (2), Content (2), Self–confident, Needy, In good spirits, Enthusiastic, Evasive, Driven, Curious, Relaxed, Frustrated, Teasing, Pushy, Quiet, Social, Bullying, Satisfied, Sad, Excited

#### Differences in QBA scores between housing condition, age class and sex

Neither the student nor the expert group gave different scores on the expressive qualities of the bonobos between the old and new enclosure on dimension 1 (students: χ² = 0.236, df = 1; *P* = 0.627; experts: χ² = 0.036, df = 1; *P* = 0.850), or dimension 2 (students: χ² = 0.126, df = 1; *P* = 0.723; experts: χ² = 0.591, df = 1; *P* = 0.442).

**Figure 1.**
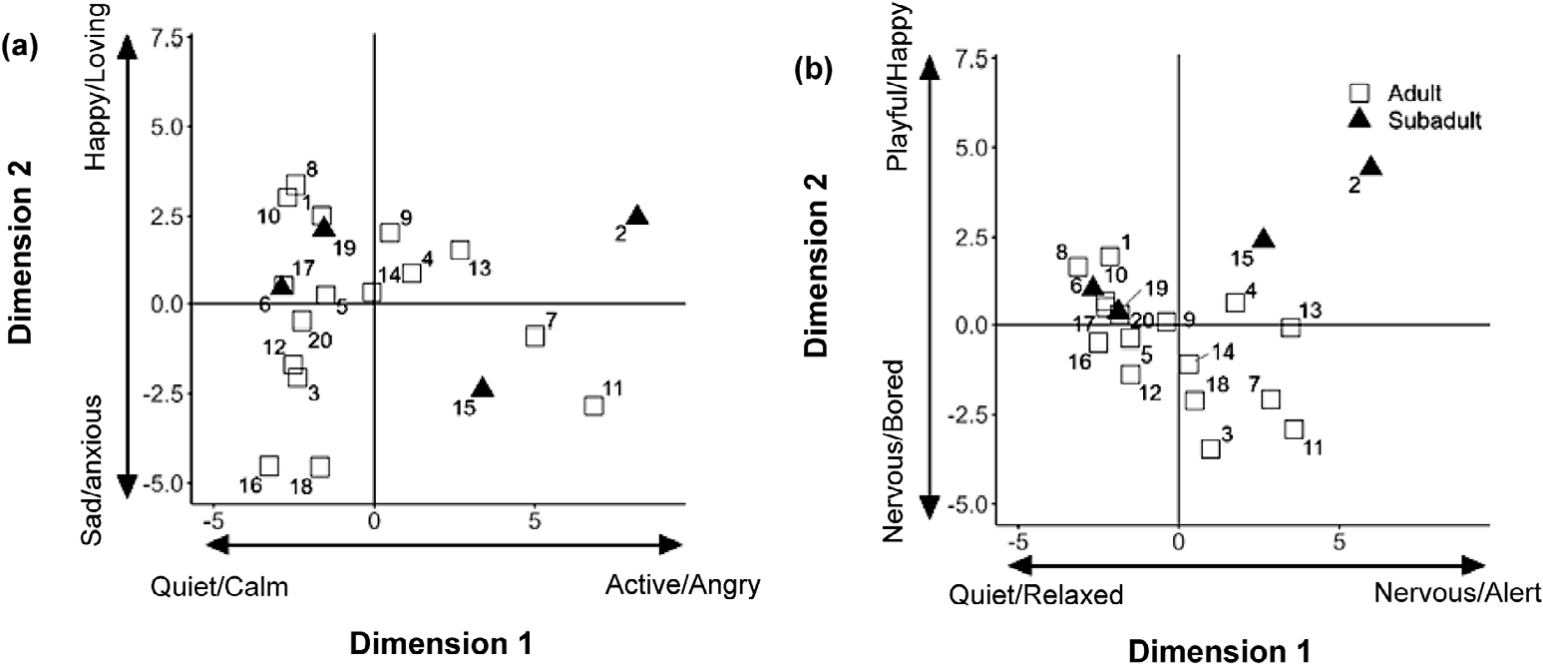
Multiple Factor Analysis (MFA) compromise scores for each of the 20 (numbered) video clips observed in Study 1 Phase 2 along the two MFA dimensions for the (a) student (n = 17) and (b) expert (n = 9) group. White squares indicate video clips of adult bonobos and black triangles subadult bonobos. The most common descriptors with the highest or lowest loadings are used to label the two dimensions.

For age class, again neither the students nor the experts gave different QBA scores for the subadults and adults on dimension 1 (students: χ² = 1.350, df = 1; *P* = 0.245; experts: χ² = 0.744, df = 1; *P* = 0.388). Additionally, the students did not score subadults differently from adult bonobos based on the QBA terms on dimension 2 (χ² = 0.365, df = 1; *P* = 0.546), whereas the experts did (χ² = 8.217, df = 1; *P* = 0.004). Specifically, experts scored subadult bonobos as more playful/happy than adults (Figure 1; *t*_18_ = 2.866; *P* = 0.010).

For sex, we found no perceived differences for students or experts on either dimension 1 (students: χ² = 2.149, df = 1; *P* = 0.143; experts: χ² = 1.119, df = 1; *P* = 0.290) or dimension 2 (students: χ² = 2.131, df = 1; *P* = 0.144; experts: χ² = 0.007, df = 1; *P* = 0.933).

#### Effects of animal-directed empathy scores

The level of empathy for animals had no influence on the representation of students and experts on dimension 1 (students: *F*_1,14_ = 2.565; *P* = 0.132; experts: *F*_1,7_ = 0.080; *P* = 0.786) or dimension 2 (students: *F*_1,14_ = 0.050; *P* = 0.827; experts: *F*_1,7_ = 0.062; *P* = 0.811).

### Study 2: Fixed List procedure

#### Inter-observer reliability

The KMO test indicated that the descriptors were suitable for the analysis, with an overall value of 0.90 for the students and 0.87 for the experts. Descriptor-specific KMO values are presented in Table S6 (see Supplementary material).

**Figure 2.**
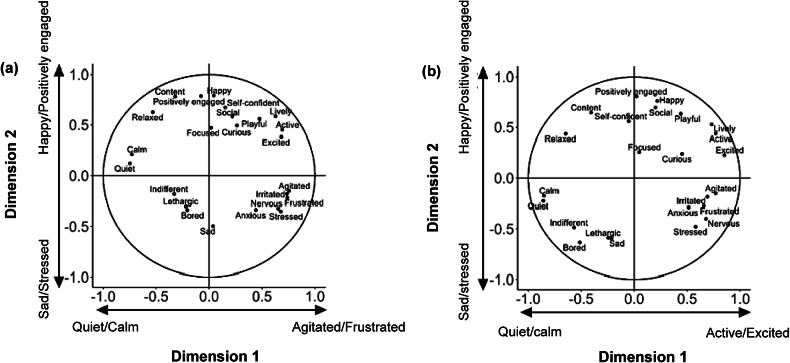
Correlation plot for the distribution of the different descriptors in Study 2 for (a) students (n = 44) and (b) experts (n = 5) alongside the two Dual Multiple Factor Analysis dimensions.

The first two dimensions of the compromise profile explained 48.7% of the variance (dimension 1 = 26.0%; dimension 2 = 22.7%). Selecting the two terms with the highest and lowest loadings on the two dimensions enabled us to characterise the dimensions. For the students, dimension 1 ranged from ‘quiet/calm’ to ‘agitated/frustrated’, and dimension 2 from ‘sad/stressed’ to ‘happy/positively engaged’. For the experts, dimension 1 ranged from ‘quiet/calm’ to ‘active/excited’, and dimension 2 from ‘sad/bored’ to ‘happy/positively engaged’. Furthermore, observer agreement across groups was good for the first dimension for students (0.717) and experts (0.764). The second dimension achieved a poor agreement for students (0.359) and moderate agreement for experts (0.586). Figure 2 shows the correlation plot of the different terms among the two dimensions for the two observer groups, separately.

Intra-class correlation analyses for separate terms were all significantly different from random expectation (Table 3; *P* < 0.001), of which students achieved poor agreement on 20 terms (ICC < 0.50; Agitated, Anxious, Bored, Content, Curious, Excited, Focused, Frustrated, Happy, Indifferent, Irritated, Lethargic, Lively, Nervous, Playful, Positively engaged, Relaxed, Sad, Self-confident and Stressed), and moderate agreement on four terms (ICC = 0.50–0.75; Active, Calm, Quiet, Social). Experts achieved poor agreement on 13 terms (Agitated, Anxious, Content, Curious, Focused, Frustrated, Irritated, Lethargic, Nervous, Relaxed, Sad, Self-confident and Stressed), moderate agreement on eight terms (Bored, Calm, Happy, Indifferent, Lively, Positively engaged, Quiet and Social) and good agreement on three terms (ICC = 0.75–0.90; Active, Excited and Playful).

**Table 3.** Inter-observer reliability on the two Dual Multiple Factor Analysis dimensions and the individual descriptors for the two observer groups (44 students and 5 experts) in Study 2

	Students				Experts			
	ICC	95% confidence interval	*F-*value (39, 1677)	*P-*value	ICC	95% confidence interval	*F-*value (39, 156)	*P-*value
*Dimension 1*	0.717	(0.627–0.808)	112.0	< 0.001	0.764	(0.662–0.851)	17.2	< 0.001
*Dimension 2*	0.359	(0.268–0.485)	25.6	< 0.001	0.586	(0.449–0.720)	8.08	< 0.001
Active	0.602	(0.500–0.716)	67.5	< 0.001	0.805	(0.716–0.879)	21.7	< 0.001
Agitated	0.385	(0.235–0.443)	28.6	< 0.001	0.329	(0.113–0.409)	3.5	< 0.001
Anxious	0.321	(0.183–0.370)	21.9	< 0.001	0.242	(0.440–0.713)	2.6	< 0.001
Bored	0.295	(0.361–0.589)	19.5	< 0.001	0.630	(0.225–0.533)	9.5	< 0.001
Calm	0.546	(0.317–0.543)	54.0	< 0.001	0.686	(0.672–0.856)	11.9	< 0.001
Content	0.305	(0.484–0.702)	20.3	< 0.001	0.356	(0.636–0.837)	3.8	< 0.001
Curious	0.224	(0.306–0.530)	13.7	< 0.001	0.406	(0.228–0.536)	4.4	< 0.001
Excited	0.414	(0.156–0.330)	32.1	< 0.001	0.771	(0.262–0.568)	17.9	< 0.001
Focused	0.240	(0.179–0.364)	14.9	< 0.001	0.386	(0.076–0.363)	4.1	< 0.001
Frustrated	0.402	(0.231–0.438)	30.5	< 0.001	0.371	(0.136–0.437)	4.0	< 0.001
Happy	0.257	(0.222–0.426)	16.2	< 0.001	0.577	(0.214–0.522)	7.8	< 0.001
Indifferent	0.220	(0.443–0.668)	13.4	< 0.001	0.546	(0.565–0.796)	7.0	< 0.001
Irritated	0.462	(0.291–0.513)	38.7	< 0.001	0.367	(0.190–0.497)	3.9	< 0.001
Lethargic	0.217	(0.178–0.363)	13.2	< 0.001	0.384	(0.181–0.487)	4.1	< 0.001
Lively	0.486	(0.169–0.350)	42.5	< 0.001	0.688	(0.243–0.550)	12.0	< 0.001
Nervous	0.274	(0.412–0.640)	17.6	< 0.001	0.383	(0.640–0.839)	4.1	< 0.001
Playful	0.464	(0.153–0.325)	39.0	< 0.001	0.812	(0.405–0.688)	22.6	< 0.001
Positively engaged	0.278	(0.151–0.322)	18.0	< 0.001	0.502	(0.241–0.548)	6.0	< 0.001
Quiet	0.586	(0.287–0.508)	63.3	< 0.001	0.744	(0.221–0.529)	15.5	< 0.001
Relaxed	0.381	(0.383–0.612)	28.1	< 0.001	0.363	(0.567–0.797)	3.9	< 0.001
Sad	0.251	(0.196–0.390)	15.8	< 0.001	0.320	(0.240–0.547)	3.4	< 0.001
Self–confident	0.252	(0.200–0.395)	15.8	< 0.001	0.199	(0.359–0.652)	2.4	< 0.001
Social	0.515	(0.214–0.415)	47.8	< 0.001	0.747	(0.499–0.754)	15.7	< 0.001
Stressed	0.316	(0.362–0.591)	21.3	< 0.001	0.270	(0.725–0.883)	2.9	< 0.001

#### Differences in QBA scores between housing condition, age class and sex

Students’ QBA scores did not show a difference between housing conditions on dimension 1 (χ² = 1.684, df = 1; *P* = 0.194) or dimension 2 (χ² = 2.38, df = 1; *P* = 0.123). For the expert observer group, there was a significant effect of housing condition on the bonobos’ scores on dimension 1 (χ² = 4.580, df = 1; *P* = 0.032), where experts scored the bonobos more ‘active/excited’ in the post-move condition compared to pre-move (*t*_38_ = 2.140; *P* = 0.039). There was no difference in how the experts scored the bonobos on dimension 2 between the two housing conditions (χ² = 0.841, df = 1; *P* = 0.359).

Additionally, for the student observer group, there was a significant effect of the bonobos’ age class on the scores on dimension 1 (χ² = 6.216, df = 1; *P* = 0.013), with students scoring adults more ‘calm/quiet’ than subadults (*t*_38_ = –2.493; *P* = 0.017). No age effect was found in the students’ scores on dimension 2 (χ² = 1.346, df = 1; *P* = 0.246). For the expert group, no significant effect was found of the bonobos’ age class on the experts’ scores on dimension 1 (χ² = 0.882, df = 1; *P* = 0.348), but was for dimension 2 (χ² = 20.879, df = 1; *P* < 0.001). That is, experts scored subadults more ‘happy/positively engaged’ than adults (*t*_38_ = 4.569; *P* < 0.001).

No significant effect of the bonobos’ sex was found for either the student or the expert group on dimension 1 (students; χ² = 0.022, df = 1; *P* = 0.883; experts: χ² = 1.430, df = 1; *P* = 0.232) or dimension 2 (students: χ² = 1.424, df = 1; *P* = 0.233; experts: χ² = 0.158, df = 1; *P* = 0.691).

#### Effects of animal-related empathy scores

A main significant effect of empathy scores was found on dimension 1 (χ² = 7.185, df = 1; *P* = 0.007), with a negative association between animal-directed empathy scores and scores on dimension 1 (*t*_39_ = –2.116; *P* = 0.041). No such effect of empathy scores was found for dimension 2 (χ² = 0.639, df = 1; *P* = 0.424).

## Discussion

### Study 1: Free Choice Profiling

In Study 1 we aimed to assess how bonobo experts and students perceived the emotional expressions of zoo-housed bonobos. We used a digital, rather than a paper-based, VAS and additionally used a different statistical approach, i.e. MFA, to analysing the FCP data which has certain advantages over GPA and is more easily accessible. Both experts and students identified two dimensions which were roughly similar in terms of their labelling. Students described dimension 1 as ranging from ‘angry/active’ to ‘quiet/calm’, and dimension 2 as ‘sad/anxious’ to ‘happy/loving’, whereas experts described dimension 1 from ‘nervous/alert’ to ‘quiet/relaxed’, and dimension 2 from ‘nervous/bored’ to ‘playful/happy’. Inter-observer reliability was adequate for both dimensions within the two groups. Moreover, the use of these dimensions was independent of animal-directed empathy levels of the observers, suggesting that variation in such empathy levels was no concern for the application of QBA on bonobos by the current observers. Additionally, we aimed to examine if observers perceived differences in these expressions based on contextual (i.e. old enclosure and new enclosure) and individual (age class, sex) factors. Observers did not perceive differences in the emotional expressions based on housing condition or between the sexes, yet experts considered subadult bonobos as more ‘playful’ and ‘happy’ than adults.

### Study 2: Fixed List procedure

In Study 2, we built upon the knowledge gained from Study 1 to develop a list of 24 fixed descriptors. Through a combined analysis of student and expert QBA scoring, we identified two dimensions that accounted for almost 50% of the variation. The student group characterised dimension 1 from ‘quiet/calm’ to ‘agitated/frustrated’, and dimension 2 from ‘sad/stressed’ to ‘happy/positively engaged’. The expert group characterised dimension 1 from ‘quiet/calm’ to ‘active/excited’, and dimension 2 from ‘sad/bored’ to ‘happy/positively engaged’. The correlation patterns of these 24 descriptors were convergent between the students and experts. Reliability in using these dimensions was generally good for both observer groups in relation to dimension 1, but lower for dimension 2. Notably, the experts showed a greater agreement on dimension 2 compared to the students’ group, consistent with our findings from Study 1.

Examining the reliability of individual descriptors, we observed overall low agreement, although the agreement was relatively higher among the experts. Nonetheless, the methodology of QBA is based upon statistically integrated dimensions in which the reliability is located, instead of within the reliability of the single descriptors. This insight underscores the need to consider broader dimensions in evaluation, but it also suggests that this information can still be of value in guiding the selection of terms for further development. Animal-directed empathy influenced observers’ scores on dimension 1, regardless of their expertise levels, with more empathic observers rating animals as more ‘quiet/calm’. In addition, it was identified that observers scored the emotional expressions of the bonobos differently based on contextual or individual factors. Both students and experts rated subadults and adults differently, although the former group allocated these differences on their respective dimension 1 and the latter on their dimension 2. Specifically, students scored adults as more ‘quiet’ and ‘calm’ compared to subadults, while experts scored subadults higher on ‘happy’ and ‘positively engaged’ than adult bonobos. Consistent with Study 1, neither observer group perceived differences between male and female bonobos. Experts also rated bonobos in the new enclosure higher on ‘active’ and ‘excited’ compared to their old enclosure, suggesting a change in the bonobos’ expressivity on this dimension linked to their novel housing condition.

### General discussion

Despite still being limited, the application of QBA in non-domesticated species is growing, especially in zoo and sanctuary settings (Patel *et al.* 2019; Pollastri *et al.* 2021; Stagni *et al.* 2022; Warner *et al.* 2022; Skovlund *et al.* 2023). Primates have thus far been underrepresented (Gartland *et al.* 2022) despite the need for quick and reliable animal-, rather than resource-based, welfare assessments in this taxon. This paper aimed to examine how human observers describe and perceive emotional expressions in bonobos. In Study 1, observers had to use their own terminology to describe bonobo expressivity and in Study 2 we applied the insights from Study 1 to develop a list of fixed descriptors. In both studies, analyses of the patterns in which the observers scored and used the descriptors revealed two dimensions that may be interpreted as dimensions of arousal and valence. The recognition of these dimensions in bonobo expressivity appeared irrespective of expertise level, as bonobo experts and students (without experience with bonobos) came up with convergent terminologies (Study 1) and used fixed terms in a similar fashion (Study 2). This is an important finding as arousal and valence are often considered as the two main dimensions of affective states in animals (Mendl *et al.* 2010).

In both studies, students and experts showed good agreement on the first dimension, but agreement was lower when scoring the second dimension. This can be partly due to the statistical analyses (i.e. [D]MFA), which aim to explain the majority of the variation in the first dimension. Alternatively, the terms linked to dimension 1 and 2 more or less reflect concepts of arousal and valence, respectively. It is possible that human observers show lower agreement in describing and assigning emotional valence to bonobos compared to levels of arousal. Recognising valence components of primate emotional expressions has proven to be difficult in other studies using static images (Maréchal *et al.* 2017; Kret & van Berlo 2021), whereas the recognition of arousal might be more widely shared among mammalian species (Greenall *et al.* 2022). The expression of emotions is highly dependent upon the context, and the recognition of emotional expressions is therefore rarely reliable on isolated cues (Ngo & Isaacowitz 2015). Although the holistic nature of QBA allows such contextual cues to be incorporated in the perception by human observers and is therefore more inclusive than for example still-images, the correct interpretation of how an animal experiences certain events or stimuli requires knowledge of the species. This likely explains why experts showed a higher agreement on the ‘valence’ dimensions than students, especially when using fixed descriptors. We purposefully provided no information regarding bonobos to the student group since we were interested in examining whether the level of experience had an effect on the development of this QBA. Agreement between the students would possibly increase when more information was forthcoming as exposure to a species enhances the recognition of expressions (Maréchal *et al.* 2017) and reliability in using fixed list descriptors (Minero *et al.* 2016).

Arousal is typically characterised by levels of activation, or the intensity of the expression, which is more readily visibly discernible and could explain why both students and experts reached good agreement on the dimension that reflects arousal. Valence, on the other hand, refers to the positivity or negativity of the emotion and requires knowledge of the species to recognise. Experience with the species has previously been shown to facilitate more accurate emotion recognition (Duijvesteijn *et al.* 2014; Menchetti *et al.* 2019; Greenall *et al.* 2022), and the higher degree of similarity in terms of experience with bonobos among the experts likely facilitated more consistent QBA scoring on the dimensions. Although the experts had knowledge about the bonobo as a species, and therefore agreed on the valence of their expressions to a certain degree, they did not work directly with the bonobos used in the videos here. We predict that people who work regularly with the individual bonobos, such as caretakers, will reach a higher agreement on the second dimension of the bonobos’ emotional expressivity as they are likely able to identify minor changes in the animals’ body language over time. Finally, we should acknowledge that our observer groups present a rather homogenous group, in the sense that they represent a W.E.I.R.D. (Western, educated, industrialised, rich and democratic) sample (Henrich *et al.* 2010), meaning that the implications of our results are potentially limited to our sample.

While expertise plays a role in the application of QBA, our study also revealed animal-directed empathy to be associated with variations in QBA usage. Individuals with higher levels of empathy for animals tended to score bonobos lower on dimension 1, irrespective of their level of expertise. Or, in other words, observers with more empathy for animals scored the bonobos as more ‘quiet’ and ‘calm’. The literature on the influence of empathy on human perception of animal emotions presents conflicting findings. Some studies suggest that participants with higher empathic scores perceive emotional expressions as more intense (Westbury & Neumann 2008; Allen-Walker & Beaton 2015), while other do not (Kujala *et al.* 2017), although this may be further modulated by experience with the species depicted (Meyer *et al.* 2014). The reason for the current observed negative correlation between empathy and scores on dimension 1 in bonobos is not clear, but since the correlation is rather weak, other factors, that we did not address, may play a larger role. Nonetheless, levels of animal-directed empathy should be taken into account when conducting QBAs, and further investigation is warranted to explore these modulating factors.

Human observers scored bonobo expressivity differently based on the enclosure within which the bonobos were housed and their age class, with no significant effect of sex. However, these effects varied between student and expert groups. Namely, experts, across both studies, gave subadult bonobos higher scores on the second dimension (i.e. more ‘playful/happy’ in Study 1, and more ‘happy/positively engaged’ in Study 2) than adults. Students instead scored adult bonobos lower on dimension 1 (i.e. more ‘quiet/calm’) than subadults. This could be explained by age-related differences in the behavioural repertoire of bonobos. For example, despite adult bonobos still engaging in social play activities (Palagi & Paoli 2007), this behaviour is more common in subadult individuals. QBA dimensions correlate with performed behaviours at the time of rating (Pollastri *et al.* 2021; Warner *et al.* 2022; Skovlund *et al.* 2023), and age-specific behavioural patterns may therefore influence the outcomes of QBA ratings. Although we did not directly correlate behaviours with the QBA scores, it is possible that these differences in behavioural patterns between adult and subadult bonobos were perceived by the observers, but that they attributed these to different emotional concepts (e.g. arousal and valence). People with experience of bonobos (e.g. caretakers or researchers) are typically more adept than the general public at distinguishing positive and negative bonobo facial expressions (Laméris, unpublished data). Hence, the expert group’s knowledge of bonobos could have facilitated recognition of behaviours as emotionally positive or negative for the animals, whereas the student group noticed levels of activity.

Observers also perceived changes in the expressivity of the bonobos based on their housing condition. Experts in Study 2 scored the bonobos as more ‘active’ and ‘excited’ in the new enclosure as compared to the old. The indoor housing conditions were more or less similar in the old and new enclosure, with the main difference in the new enclosure being the presence of two outdoor areas as opposed to one. Hence, while the two bonobo groups previously had to alternate for outdoor access, in the new enclosure they were able to access their own outdoor area simultaneously. Outdoor access has previously been proven to be beneficial for the behaviour and welfare of zoo-housed primates (Videan *et al.* 2005; Honess & Marin 2006; Pines *et al.* 2007; Kurtycz *et al.* 2014; Laméris *et al.* 2021), and this could explain the differences in the expressivity of the bonobos observed. This provides valuable information regarding possible welfare implications of changing zoo enclosures.

## Animal welfare implications and conclusion

In conclusion, human observers with varying levels of expertise perceived similar dimensions of emotional expressivity in bonobos. The reliability of these dimensions, however, increased with experience, especially when scoring differences in valence. Experience thus allowed for more nuanced perceived differences in the emotional expressivity based on contextual and individual factors. We recommend incorporating knowledge from people familiar with the individual animals in question and work regularly with them, such as care staff, who are more able to notice subtle changes in the emotional expressivity of the animals over time. No one single indicator can be considered exhaustive to evaluate the welfare of an animal, and QBA is no exception. Welfare is a complex, multifaceted concept that requires multiple indicators for accurate assessment. The QBA developed and tested in the current study could potentially be incorporated into welfare assessment protocols if further (cross-)validation against other well-established welfare measures proves its value.

## Supporting information

Laméris et al. supplementary material Laméris et al. supplementary material

## Data Availability

The data that support the findings of this study are available from DWL upon request.

## Supplementary material

The supplementary material for this article can be found at http://doi.org/10.1017/awf.2024.29.
