# Supplementary material for: Qualitative Behavioural Assessment of bonobo emotional expressivity across observer groups and zoo housing environments
Source: Anim Welf. 2024 May 30;33:e28. doi: 10.1017/awf.2024.29 (PMC11140493; doi:10.1017/awf.2024.29)
Supplement: Laméris et al. supplementary material [file S0962728624000290sup001.pdf]

General

Table S1. Distribution of 30-s video clips across subject, sex, age class, and housing condition

Subject	Sex	Age class	Old enclosure	New enclosure
Animal 1	Female	Adult	15	13
Animal 2	Female	Adult	10	13
Animal 3	Female	Adult	12	19
Animal 4	Female	Adult	17	28
Animal 5	Female	Adult	8	14
Animal 6	Male	Adult	11	16
Animal 7	Male	Adult	7	14
Animal 8	Male	Adult	5	14
Animal 9	Male	Subadult	16	21
Animal 10	Male	Subadult	10	15

Table S2. Demographics of human participants in Study 1 and Study 2

	Study 1		Study 2	
**Experience**				
Student	17	65.4%	38*	88.4%
Expert	9	34.6%	5	11.6%
**Pet ownership**				
Yes, present	23	88.5%	39	90.7%
Yes, past	3	11.5%	4	9.3%
No	0	0.0%	0	0.0%
**Zoo visits**				
0 visits/year	6	23.1%	13	30.2%
1–5 visits/year	12	46.2%	24	55.8%
6–10 visits/year	2	7.7%	1	2.3%
+10 visits/year	6	23.1%	5	11.6%
**Work experience (only students)**				
Yes, present	12	70.6%	28	73.7%
With primates	1	5.9%	2	5.3%
Yes, past	4	23.5%	6	15.8%
With primates	0	0.0%	0	0.0%
No	0	0.0%	2	5.3%

* Six students did not fully complete the survey and were not included here

Table S3. Clustering of Animal Empathy statements based on Ward Hierarchical Clustering analysis

**Cluster 1 – 'Empathic'**
5. Sad films about animals often leave me with a lump in my throat.
7. It makes me sad to see an animal on its own in a cage.
9. A friendly purring cat almost always cheers me up.
10. It upsets me when I see helpless old animals.
13. I get very angry when I see animals being ill-treated.
15. Pets have a great influence on my moods.
17. I enjoy feeding scraps of food to the birds.
18. Seeing animals in pain upsets me.
21. I would always try to help if I saw a dog or puppy that seemed to be lost.
22. I hate to see birds in cages where there is no room for them to fly about.
23. It upsets me to see farm animals in lorries going to slaughter.
26. The thought of calves being reared in veal crates really makes me feel sad.
27. I hate seeing pictures of animals used in scientific experiments.
**Cluster 2 – 'Apathic'**
1. So long as they're warm and well fed, I don't think zoo animals mind being kept in cages.
2. Often cats will meow and pester for food even when they are not really hungry.
3. It upsets me to see animals being chased and killed by lions in wildlife programmes on TV.
4. I get annoyed by dogs that howl and bark when they are left alone.
6. Animals deserve to be told off when they're not behaving properly.
8. People who cuddle and kiss their pets in public annoy me.
11. Dogs sometimes whine and whimper for no real reason.
12. Many people are over-affectionate towards their pets.
14. It is silly to become too attached to one's pets.
16. Sometimes I am amazed how upset people get when an old pet dies.
19. People often make too much of the feelings and sensitivities of animals.
20. I find it irritating when dogs try to greet me by jumping up and licking me.
24. It's silly to worry about how farm animals feel.
25. People are too concerned about the suffering of laboratory rats and mice.

Study 1: Free Choice Profiling

Table S4. Descriptions of the videos used in Study 1, Phase 1 of the Free Choice Profiling procedure

Video	Short description
01	Bonobo resting
02	Bonobo autogrooming
03	Bonobos A and B vocalising, C charging and hitting glass towards the visitors and scratching
04	Bonobo performing solitary play
05	Bonobo mother interacting with infant
06	Bonobo A approaches B and C and starts displaying
07	Bonobo swinging through flexible structures
08	Bonobos A and B engaging in genito-genital rubbing, C harasses
09	Allogrooming among four bonobos
10	Bonobo hit on glass, then starts feeding on browse
11	Bonobo A grooms B, B embraces A, B leaves and reaches for C
12	Genito-genital rubbing between bonobo A and B, C harasses A, and A and C perform parallel display. D undirected display, and E and F perform short charge
13	Bonobo tries to open a coconut and succeeds
14	Bonobo A follows B, B displaces
15	Four bonobos gentle playing
16	Two bonobos playing with object
17	Bonobo looking through door to other group, sticking fingers through holes
18	Bonobo sits, then moves and rough scratches
19	Bonobo A sits, then vocalises and short charge to B
20	Bonobo A grooming B, A pushes away C. Bonobo D is resting and eating

The order of the videos in this table is as presented to the observer with the intention to create contrast between the content of the videos to stimulate observers to think of different terms.

Table S5. Unique descriptors used in Study 1 Phase 1 in Dutch and their English translation

	Dutch term	English term		Dutch term	English term
1	Aandacht zoekend	Attention-seeking	45	Geamuseerd	Amused
2	Aandachtig	Attentive	46	Geconcentreerd	Concentrated
3	Aanstellend	Petty	47	Gedreven	Driven
4	Actief	Active	48	Geërgerd	Annoyed
5	Affectief	Affectionate	49	Gefocust	Focused
6	Afgeleid	Distracted	50	Gefrustreerd	Frustrated
7	Afwachtend	Waiting	51	Geïnteresseerd	Interested
8	Afwerend	Rejecting	52	Geïntimideerd	Intimidated
9	Afwezig	Absent	53	Geïrriteerd	Irritated
10	Agressief	Aggressive	54	Gelaten	Relieved
11	Alert	Alert	55	Geliefd	Loved
12	Angstig	Anxious	56	Gemak	Comfortable
13	Antisociaal	Anti-social	57	Genietend	Enjoying
14	Apathisch	Apathetic	58	Gepassioneerd	Passionate
15	Avontuurlijk	Adventurous	59	Gerust	At Ease
16	Baldadig	Reckless	60	Geschokt	Shocked
17	Bang	Afraid	61	Geschrokken	Scared
18	Bazig	Bossy	62	Gespannen	Tense
19	Bedachtzaam	Pensive	63	Gestimuleerd	Stimulated
20	Bedreigd	Threatened	64	Gestoord	Disturbed
21	Behoeftig	Needy	65	Gestrest	Stressed
22	Behulpzaam	Helpful	66	Gewijzigd	Altered
23	Beschermend	Protective	67	Goedgezind	In good spirits
24	Besluiteloos	Undecided	68	Grenzen aftastend	Exploring Boundaries
25	Beweeglijk	Mobile	69	Hardhandig	Heavy Handed
26	Bezitterig	Possessive	70	Hebberig	Greedy
27	Bezorgd	Concerned	71	Helpend	Helping
28	Boos	Angry	72	Hevig	Fierce
29	Buitengesloten	Excluded	73	Hongerig	Hungry
30	Content	Content	74	Impulsief	Impulsive
31	Defensief	Defensive	75	In aandacht nood	In Need Of Attention
32	Dominant	Dominant	76	Intens	Intense
33	Doorzettend	Persistent	77	Irritant	Annoying
34	Dreigend	Threatening	78	Jaloers	Jealous
35	Druk	Busy	79	Jeukend	Itchy
36	Dwang	Compulsive	80	Kalm	Calm
37	Dwingend	Coercive	81	Knuffelig	Cuddly
38	Eenzaam	Lonely	82	Kordaat	Resolute
39	Eetlustig	Appetitive	83	Lastig	Difficult
40	Energierijk	Energetic	84	Levendig	Lively
41	Enthousiast	Enthusiastic	85	Lief	Sweet
42	Exploratief	Explorative	86	Liefhebbend	Loving
43	Explosief	Explosive	87	Loops	In Heat
44	Geagiteerd	Agitated	88	Lui	Lazy

Table S5. continued

	Dutch term	English term
89	Luid	Loud
90	Luidruchtig	Noisy
91	Lustig	Lustful
92	Moe	Tired
93	Nerveus	Nervous
94	Niet onder de indruk	Unimpressed
95	Nieuwsgierig	Curious
96	Nood aan vrijheid	In need of freedom
97	Observerend	Observant
98	Obsessief	Obsessive
99	Oncomfortabel	Uncomfortable
100	Onderdanig	Submissive
101	Onderzoekend	Inquisitive
102	Ongeduldig	Impatient
103	Ongeïnteresseerd	Uninterested
104	Ongelukkig	Unhappy
105	Ongemakkelijk	Uneasy
106	Ongerust	Worried
107	Onhandig	Clumsy
108	Onoplettend	Inattentive
109	Ontdekkend	Discovering
110	Ontevreden	Dissatisfied
111	Ontspannen	Relaxed
112	Ontwijkend	Evasive
113	Onverstoord	Undisturbed
114	Onwetend	Ignorant
115	Onzeker	Insecure
116	Opdringerig	Pushy
117	Opeisend	Demanding
118	Opgejaagd	Hunted
119	Opgewekt	Cheerful
120	Opgewonden	Excited
121	Oplettend	Watchful
122	Paniek	Panicking
123	Paniekerig	Panicky
124	Pesterig	Bullying
125	Plagerig	Teasing
126	Plezier maken	Having fun
127	Plots	Brusque
128	Prikkelbaar	Irritable
129	Psychopathisch	Psychopathic
130	Rusteloos	Restless
131	Rustig	Quiet

	Dutch term	English term
132	Slaperig	Drowsy
133	Sociaal	Social
134	Speels	Playful
135	Suf	Dull
136	Terughoudend	Reluctant
137	Terugtrekkend	Withdrawn
138	Timide	Timid
139	Toegankelijk	Accessible
140	Toegeeflijk	Permissive
141	Traag	Slow
142	Treurig	Sad
143	Troostend	Comforting
144	Trots	Proud
145	Trouw	Loyal
146	Uitdagend	Challenging
147	Van streek	Upset
148	Vastberaden	Determined
149	Verbaasd	Surprised
150	Verkennend	Exploring
151	Verlegen	Shy
152	Vermijdend	Avoidant
153	Verveeld	Bored
154	Verwachtingsvol	Expectant
155	Verward	Confused
156	Verzorgend	Nurturing
157	Vijandig	Hostile
158	Voldaan	Satisfied
159	Voorzichtig	Careful
160	Vreesachtig	Fearful
161	Vriendelijk	Friendly
162	Vrolijk	Happy
163	Waakzaam	Vigilant
164	Waarschuwend	Warning
165	Wantrouwig	Suspicious
166	Zeker	Confident
167	Zelf-kalmerend	Self-Soothing
168	Zelfzeker	Self-confident
169	Zorgeloos	Carefree
170	Zorgend	Caring

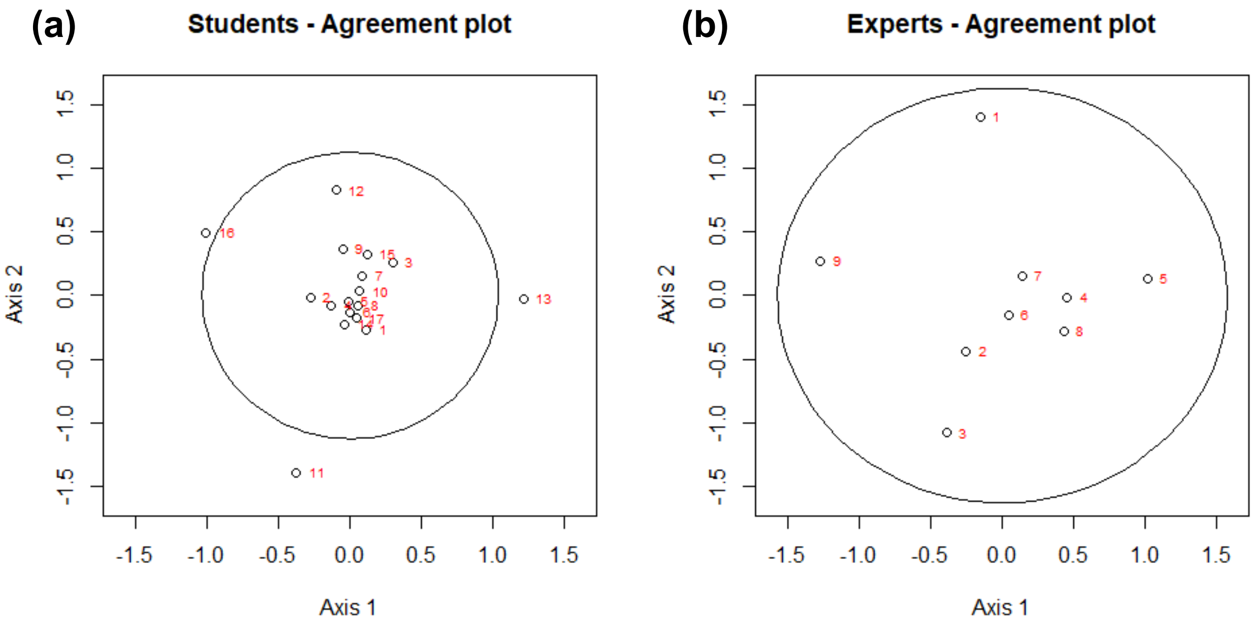

Figure S1. Observer plots for (a) students and (b) experts in Study 1. Numbers represent individual observers and the circle represents the 95% confidence ellipse.

Study 2: Fixed List procedure

Table S6. Kaiser-Meyer-Olkin (KMO) test results for Study 2

**KMO - Student**		**KMO - Expert**	
Overall	0.90	Overall	0.87
Active	0.92	Active	0.90
Anxious	0.85	Anxious	0.86
Happy	0.91	Happy	0.85
Irritated	0.87	Irritated	0.83
Excited	0.95	Excited	0.94
Quiet	0.87	Quiet	0.88
Frustrated	0.88	Frustrated	0.82
Curious	0.88	Curious	0.84
Self-confident	0.93	Self-confident	0.76
Stressed	0.90	Stressed	0.81
Content	0.90	Content	0.86
Calm	0.87	Calm	0.90
Agitated	0.94	Agitated	0.91
Sad	0.85	Sad	0.85
Focused	0.83	Focused	0.66
Social	0.94	Social	0.87
Indifferent	0.86	Indifferent	0.92
Lethargic	0.85	Lethargic	0.82
Relaxed	0.93	Relaxed	0.91
Lively	0.93	Lively	0.90
Nervous	0.92	Nervous	0.88
Positively engaged	0.92	Positively engaged	0.87
Bored	0.81	Bored	0.90
Playful	0.90	Playful	0.87
